# Influence of Dietary Factors on *Helicobacter pylori* and CagA Seroprevalence in Bulgaria

**DOI:** 10.1155/2017/9212143

**Published:** 2017-06-04

**Authors:** Daniel Yordanov, Lyudmila Boyanova, Rumyana Markovska, Juliana Ilieva, Nikolay Andreev, Galina Gergova, Ivan Mitov

**Affiliations:** ^1^Department of Medical Microbiology, Medical University of Sofia, Sofia, Bulgaria; ^2^Department of Social Medicine and Healthcare Management, Medical University of Sofia, Sofia, Bulgaria; ^3^Centre of Transfusion Hematology, “St. Anna” University Hospital, Sofia, Bulgaria

## Abstract

The aim of this study was to assess the association between some dietary factors and prevalence of *H. pylori* infection or strain virulence in 294 adult asymptomatic blood donors. *Methods*. Seroprevalence was evaluated using ELISA. Logistic regression was used. *Results*. Anti-*H. pylori* IgG prevalence was 72.4%, and CagA IgG seroprevalence was 49.3%. In the multivariate analyses, the frequent (>5 days per week) honey consumption was associated with both reduced *H. pylori* seroprevalence OR, 0.68 with 95% confidence interval (CI), 0.473–0.967 and reduced CagA IgG seroprevalence OR, 0.65 with 95% CI, 0.486–0859. Frequent (>5 days per week) yoghurt consumption also was associated with lower *H. pylori* virulence of the strains (CagA IgG OR, 0.56 with 95% CI, 0.341–0.921). Smoking and consumption of the other dietary factors resulted in no significant differences in the prevalence of *H. pylori* IgG and CagA IgG within the subject groups. *Conclusion*. To the best of our knowledge, this is the first report revealing reverse associations between honey or yoghurt consumption and CagA IgG prevalence as well as between frequent honey consumption and lower prevalence of the *H. pylori* infection. Regular honey and yoghurt consumption can be of value as a supplement in the control of *H. pylori* therapy.

## 1. Introduction

Optimal treatment of *Helicobacter pylori* infection still remains a significant problem, although it has been extensively studied. Standard regimens for *H. pylori* eradication include a proton pump inhibitor and usually two antibiotics. Yet, recently, these regimens often fail due to antibiotic resistance or low patient compliance [1]. So, there is a growing need for new drugs such as nonantibiotic agents (NAAs) or optimized treatment regimens.

Anti-*H. pylori* activity has been detected in vitro by many NAAs, such as lactobacilli and other probiotics, extracts from green tea, garlic, propolis, honey, plant oils, and resveratrol, and has been associated with their antiadhesive properties and bacteriostatic and bactericidal activities against *H. pylori*, including antibiotic-resistant strains, anti-inflammatory and anticancer effects, improvement of *H. pylori* eradication, and decrease of side effects of the standard regimens [2]. However, it should be noted that the in vitro activity against *H. pylori* does not always indicate the in vivo effect of the NAAs [3]. Furthermore, using different methods and various sources of the same agent may result in discrepancy between results [4].

*H. pylori cagA* pathogenicity island that encodes type IV secretion system has been associated with severe diseases [5]. CagA protein, injected to gastric epithelial cells via a secretion system, possesses both cytotoxic and proinflammatory activities. The protein is highly immunogenic, and CagA antibody detection shows association with the development of peptic ulcer and gastric cancer [5].

The aim of the present study was to determine whether specific dietary factors influence *H. pylori* positivity or the pathogen's virulence by assessing the prevalence of *H. pylori* immunoglobulin G (IgG) and CagA IgG antibodies in asymptomatic blood donors.

## 2. Materials and Methods

In total, 294 serum samples were collected from asymptomatic blood donors aged 18–69 years at the Department of Transfusion Hematology in “St. Anna” University Hospital in Sofia. All subjects signed informed consents and filled a questionnaire with data for smoking and consumption of alcohol, yoghurt, green tea, coffee, raw garlic and raw onion, honey bee, fresh fruits and vegetables, red wine, and olive oil. For smoking, the frequency was determined by the “number of cigarettes smoked daily” (nonsmokers, ≤20 cigarettes/day, and >20 cigarettes/day). For the other dietary factors, the frequency of consumption was considered (no consumption, consumption ≤5 days per week, or >5 days per week).

The sera were separated and stored at −30°C until assayed. A single specimen per subject was examined. *H. pylori* and CagA IgGs were detected using commercial kits for enzyme-linked immunosorbent assay (ELISA, Euroimmun, Medizinische Laboratordiagnostika AG, Germany), including anti-*Helicobacter pylori* ELISA (IgG) and anti-*Helicobacter pylori* CagA ELISA (IgG). For every batch, sera from all subject subgroups were examined. Testing was performed according to the manufacturer's instructions.

Statistical Analysis. Statistical analysis was performed using SPSS 15.0 statistical software. A *χ*^2^ test of independence was used to compare the following variables of interest: age, sex, and the presence of smoking and various dietary factors such as consumption of alcohol, coffee, fresh fruit, fresh vegetables, green tea, honey, olive oil, raw garlic, raw onion, and yogurt. Logistic regression was used to select significant predictor variables and to estimate the odds ratios (ORs) of these variables and, if possible, to predict outcomes. Only the age was detected as an important confounder in multivariate models. Receiver operating characteristic (ROC) curves were performed to assess the ability of the model of logistic regression to predict a positive event.

## 3. Results

Overall prevalence of *H. pylori* IgG among the 294 subjects was 72.4% (213 cases), and CagA IgGs were found in 145 subjects (49.3%). Three sera showed positive result for IgG CagA and negative for IgGHp. The detailed results are shown in Table 1.

The univariate analyses showed that frequent (>5 days per week) honey bee intake was linked to significantly lower *H. pylori* (60.8% versus 76.4% in the other subjects, *p* = 0.01) and CagA IgG seroprevalence (35.1% versus 54.1%, *p* = 0.005) (Table 1). Furthermore, frequent (>5 days per week) yoghurt consumers had significantly lower CagA IgG seroprevalence (40.4%) compared to the rest of the subjects (54.6%, *p* = 0.016) (Table 1).

**Figure 1:**
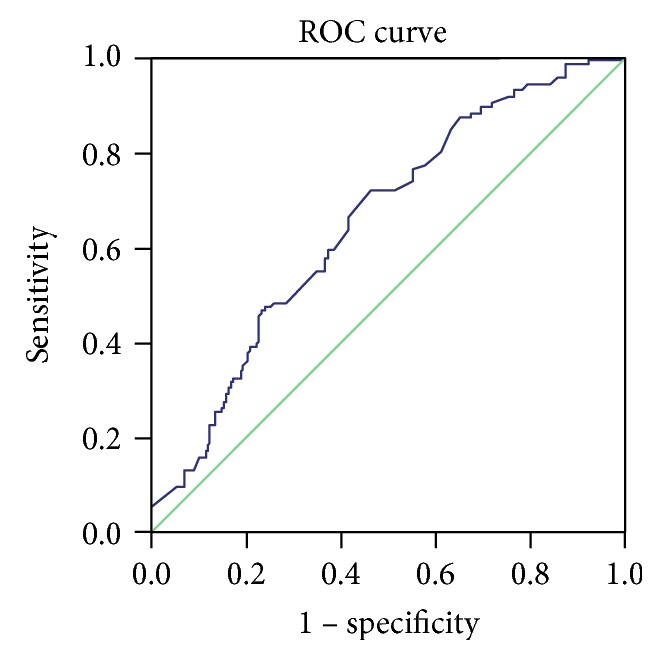
ROC curve for logistic regression model for risk factors (age and yoghurt and honey consumption) for CagA IgG prevalence.

Logistic regression analysis showed a lower risk of infection with virulent CagA-positive *H. pylori* in frequent honey consumers >5 days/week (OR, 0.65; 95% confidence interval (CI), 0.486–0.859) and yoghurt consumers (OR, 0.56; 95% CI, 0.341–0.921) compared with that in the other subjects (Table 2). The respective ROC curve showed that the area under the curve (AUC) for anti-CagA IgG was 0.654 (SEM 0.032, *p* < 0.001, 95% CI 0.592–0.717) when age and yoghurt and honey intake were taken into account (Figure 1).

**Figure 2:**
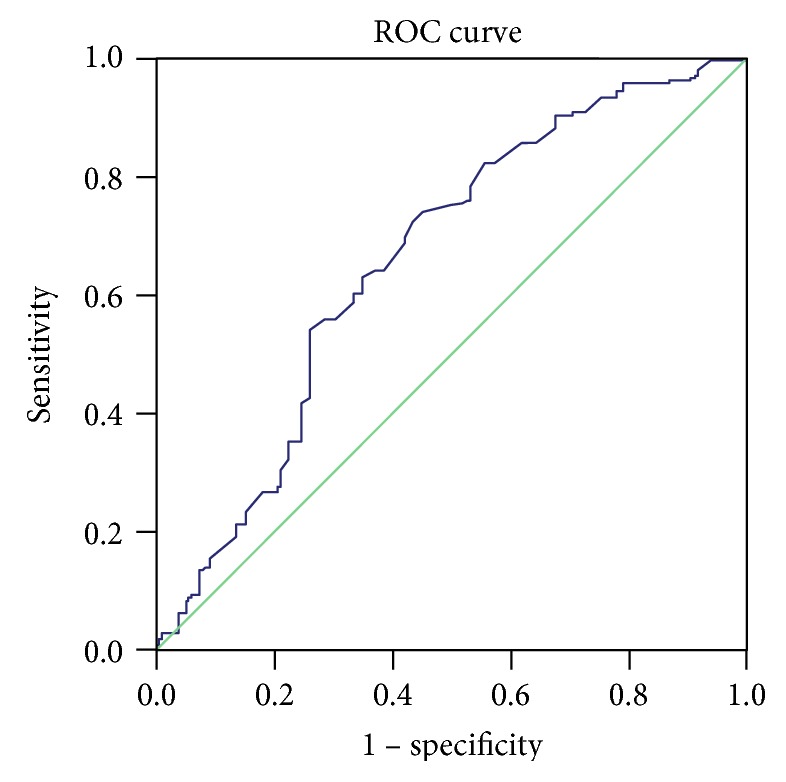
ROC curve for logistic regression model for risk factors (age and honey consumption) for *H. pylori* IgG prevalence.

Logistic regression analysis showed a lower risk of *H. pylori* infection in frequent honey consumers >5 days/week (OR, 0.68; 95% (CI), 0.473–0.967) compared with that in the other subjects (Table 3). The respective ROC curve showed that the area under the curve (AUC) for *H. pylori* IgG was 0.662 (SEM 0.038, *p* < 0.001, 95% CI 0.588–0.736) when age and honey intake were taken into account (Figure 2).

The dietary factor consumption except for the honey and the yogurt resulted in no significant differences in the prevalence of *H. pylori* IgG and CagA IgG within the subject groups. In univariate analyses, smoking was associated with an increase in anti-CagA IgG prevalence; however, the logistic regression did not confirm this association.

## 4. Discussion

The results in the present study showed that *H. pylori* IgG seroprevalence was significantly lower among the people who consumed honey regularly (>5 days per week).

The anti-*H. pylori* activity of honey has been reported previously. The effect of honey is due to its pH, hydrogen peroxide, osmotic effect, and other substances [6]. Ndip et al. [7] have evaluated the activities of Mountain honey from Cameroon, Capilano and Manuka honeys from New Zealand, South African honeys, and Eco-Honey from Kenya. Of these, mountain honey and Manuka honey have been the most active anti-*H. pylori* agents [7]. The concentration of Mountain honey and South African honeys which demonstrated the best effect against *H. pylori* has been by 75% *v*/*v* [6]. The effect of the Manuka honey has been associated with a unique Manuka factor (UMF) with an activity corresponding to that of 20% *w*/*v* phenol [8, 9] demonstrated anti-inflammatory properties of the Manuka honey due to decrease in interleukin 8 (IL-8) release from *H. pylori*-infected cells [9]. However, in a study on wound healing, a 5.8 kDa factor of the Manuka honey interacting with Toll-like receptor 4 (TLR4) has been found to activate the production of the proinflammatory mediator, tumor necrosis factor alpha (TNF-*α*) [8]. That is why the activities of the Manuka honey should be considered carefully.

On the other hand, a study in Oman demonstrated the antibacterial properties of various brands of honey on *H. pylori* isolates, yet the results showed that there was no synergy between honey and amoxicillin or clarithromycin used in the treatment of *H. pylori* gastritis and duodenal ulcer [10].

In search for active molecules against *H. pylori*, Manyi-Loh et al. [11] have performed thin layer chromatography on Goldcrest (GC) n-hexane extract. A total of 16 volatile compounds with known antimicrobial and antioxidant properties, such as alcohol, ketone, aliphatic acid, benzene compound, hydrocarbon, furan, and pyran derivatives, have been identified, and the best antibacterial activity has been demonstrated by the fraction GCF3 (5 mg/ml) [11].

In Bulgaria, anti-*H. pylori* activity of other bee products such as propolis has been studied in vitro [12]. Growth of all *H. pylori* strains has been inhibited by 90 *μ*l of 30% ethanol extract of Bulgarian propolis per well in agar well diffusion method. By agar dilution method, 100 and 300 *μ*g/ml propolis have inhibited 57.1% and 76.2% of strains, respectively [12]. Bulgarian propolis has demonstrated a dose-dependent activity against most *H. pylori* strains tested [12]. However, Coelho et al. [13] found that the use of Brazilian propolis for just one week provided only transitory *H. pylori* inhibition in some patients. Nevertheless, it is well known that even antibiotic monotherapy leads to *H. pylori* eradication in only 10–50% of the cases [2]. NAAs can have better effects when consumed for a long time (several months) to obtain optimal activity. In a recent study, 50.6% of the Bulgarian gastroenterology patients consuming honey ≥1 days/week were *H. pylori* positive (evaluated by ^13^C urea breath test) versus 70.8% of the other patients [14]. The present study supports this observation and underlined that frequent continuous consumption of honey (>5 days every week and, supposedly, for a long time period) was associated with lower *H. pylori* IgG prevalence.

It is also fascinating that the present study demonstrated a strong association between lower CagA IgG prevalence and frequent honey consumption (>5 days weekly). Interestingly, three subjects were *H. pylori* IgG negative but anti-CagA positive. There are single cases of *H. pylori* infection clearance by regular antibiotic use for other infections [15]. However, according to Japanese authors, CagA antibody-positive people (IgG antibody positive and IgG negative) were at high risk for gastric cancer [16].

The present results show that, beside the lower risk of being infected with *H. pylori*, the people who frequently consume honey have a better protection against the infection with highly virulent cytotoxin-producing *H. pylori* strains. To the best of our knowledge, such a negative association between honey consumption and CagA IgG seroprevalence has never been reported so far, and therefore, the exact mechanisms should be further evaluated.

As the presence of CagA antibodies has been associated with the development of peptic ulcer and gastric cancer [5], a honey containing diet may become an easy method to reduce the prevalence of *H. pylori* infection within the population, as well as to reduce the incidence of severe gastric diseases. Yet, the significance of honey intake for the treatment of *H. pylori*-infected patients (alone or as an addition to the antibiotic-containing regimens) needs to be further investigated.

Many studies have reported anti-*H. pylori* activity of the lactobacilli*. Lactobacillus* spp. survives in the stomach longer than most of the bacteria due to their acid resistance. The anti-*H. pylori* effects comprise suppression of *H. pylori* urease activity; adhesion to gastric epithelial cells; secretion of short-chain fatty acids, for example, propionic, acetic, formic and, especially, lactic acids, as well as autolysins, bacteriocins, and bacteriocin-like or other active substances such as hydrogen peroxide, proteinases, exopolysaccharides, and cell wall components; inhibition of *H. pylori*-induced IL-8 release; and so forth [3, 17, 18]. Importantly, the probiotics could downregulate the *H. pylori* production of virulence factors [19]. For instance, *L. paraplantarum* strains have been found to reduce *cagA*+ strain adherence to gastric cell lines [20].

Lactobacilli may exhibit bactericidal anti-*H. pylori* activity, and they also block colonization and reinfection, as they inhibit the adhesion of *H. pylori* to the gastric cells. That is why lactobacilli are often included in treatment regimens for eradication of *H. pylori* [3]. However, the in vitro and in vivo activities of the probiotics may differ and the activity of the lactobacilli is extremely strain specific [17, 21]. Boyanova et al. [22] have reported that all studied *Lactobacillus delbrueckii* subsp. *bulgaricus* strains have inhibited a number of *H. pylori* strains, including those resistant to antibiotics; however, the activity of the lactobacilli has been highly strain dependent [22].

As lactobacilli are a major component in yoghurt, the present study investigated the frequency of yoghurt consumption and *H. pylori* IgG prevalence. Surprisingly, unlike Ornellas et al. [23], the present study did not find lower *H. pylori* seroprevalence among people who consume yoghurt.

Ornellas et al. [23] found protective effects of eating yoghurt more than once per week in a Mexican population. However, in the present study, only 19 persons (6.5%) stated that they do not eat yoghurt. Besides, in Bulgaria, there is a great variety (about 80) of yoghurt brands which may quite differ in respect to the concentration and the properties of the lactobacilli. That is why a further and more detailed study is necessary in order to obtain better knowledge on this topic.

It is important, however, that the present study found an association between the frequent (>5 days per week) consumption of yoghurt and the lower CagA IgG seroprevalence. This fact shows that even if the frequent yoghurt consumption does not reduce the prevalence of *H. pylori* infection, it can provide protection against the infection with highly virulent CagA-positive *H. pylori* strains.

There were not significant differences between both *H. pylori* IgG and CagA IgG prevalence and the consumption of the other dietary/lifestyle factors such as smoking and consumption of alcohol, green tea, coffee, raw garlic and raw onion, fresh fruits and vegetables, red wine, and olive oil. Similar to the current study, Zhu et al. [24] have found no differences between *H. pylori* prevalence and the consumption of dietary factors such as fruit and vegetable, onion, garlic, and wine consumption and tobacco smoking [24]. On the other hand, Łaszewicz et al. [25] have found that tobacco smoking and alcohol drinking were associated with higher *H. pylori* seroprevalence, while Ansari et al. [26] have found that green tea drinking is a factor that can decrease the prevalence of *H. pylori* infection. Although fruits and vegetables do not reduce Hp infection, their consumption may reduce gastric cancer as it has been shown in another study [27]. Some authors reported that fast food such as sausages and hamburgers and low intake of fruits and vegetables are correlated with high incidence of *H. pylori* infections [28].

Briefly, this study revealed for the first time that the frequent consumption of honey bee is associated with lower seroprevalence of *H. pylori* infection. Furthermore, the frequent consumption of honey or yoghurt has never before been reported to be associated with lower CagA IgG prevalence. Therefore, a diet including a regular intake of honey and yoghurt could provide better protection against infection with highly virulent *H. pylori* CagA^+^ strains that increase the incidence of severe diseases like peptic ulcers and gastric cancer.

## Figures and Tables

**Table 1:** *H. pylori* IgG and CagA IgG seroprevalence in 294 asymptomatic Bulgarian blood donors according to the consumption of some dietary factors.

Dietary factor	Subjects evaluated	*H. pylori* IgG positive		*p* value^2^ (*χ*^2^)	CagA IgG positive		*p* value^2^ (*χ*^2^)
	Number	Number	%		Number	%	
Age^1^							
18–30	75	40	53.3	**0.001 (18.702)**	24	32.0	**0.006 (12.62)**
31–40	75	58	77.3		39	52.0	
41–50	75	59	78.7		42	56.0	
51–69	69	56	81.2		40	58.0	
Sex^1^							
Male	218	150	68.8	**0.018 (5.603)**	101	46.3	0.082 (3.01)
Female	76	63	82.9^2^		44	57.9	
Subject's level of education^1^							
Elementary	43	37	86.0	0.097 (4.669)	27	62.8	0.105 (4.508)
Secondary	178	125	70.2		87	48.9	
Higher	73	51	69.9		31	42.5	
Profession^1,3^ (*n* = 283)							
Physical work	184	136	73.9	0.449 (0.573)	97	52.7	0.07 (3.292)
Intellectual work	99	69	69.7		41	41.4	
Smoking							
No	116	84	72.4	0.701 (0.769)	56	48.3^2^	**0.019 (3.271)**
≤20 cigarettes/day	161	115	71.4		77	47.8	
>20 cigarettes/day	17	14	82.4		12	70.6	
Alcohol							
No	85	62	72.4	0.701 (0.712)	44	51.8	0.657 (0.84)
≤5 days/week	163	120	73.2		81	67.5	
>5 days/week	46	31	67.4		20	43.5	
Coffee							
No	35	21	60.0	0.203 (3.194)	13	37.1	0.296 (2.433)
≤5 days/week	30	23	76.7		16	53.3	
>5 days/week	229	169	73.8		116	50.7	
Fresh fruits							
No	13	10	76.9	1.000 (0.111)	9	69.2	0.125 (4.153)
≤5 days/week	70	51	72.9		39	55.7	
>5 days/week	211	152	72.0		97	46.0	
Fresh vegetables							
No	7	4	57.1	0.483 (1.502)	5	71.4	0.149 (3.81)
≤5 days/week	90	68	75.6		50	55.6	
>5 days/week	197	141	71.6		90	45.7	
Green tea							
No	221	162	73.3	0.531 (1.356)	112	50.7	0.595 (1.039)
≤5 days/week	55	40	72.7		26	47.3	
>5 days/week	18	11	61.1		7	38.9	
Honey							
No	95	69	72.6	**0.019 (7.877)**	52	54.7	**0.018 (7.988)**
≤5 days/week	125	99	79.2		67	53.6	
>5 days/week	74	45	60.8^2^		26	35.1^2^	
Olive oil							
No	171	126	73.7	0.161 (3.658)	87	50.8	0.285 (2.51)
≤5 days/week	70	54	77.1		37	52.9	
>5 days/week	53	33	62.3		21	39.6	
Raw garlic							
No	63	46	73.0	0.967 (0.067)	37	58.7	0.241 (2.842)
≤5 days/week	165	120	72.7		77	46.7	
>5 days/week	66	47	71.2		31	47.0	
Raw onion							
No	65	44	67.7	0.389 (1.886)	33	50.8	0.964 (0.072)
≤5 days/week	153	116	75.8		75	49.0	
>5 days/week	76	53	69.8		37	48.7	
Red wine							
No	132	95	72.0	0.396 (1.853)	69	52.3	0.631 (0.92)
≤5 days/week	109	83	76.1		52	47.7	
>5 days/week	53	35	66.0		24	43.4	
Yoghurt							
No	21	14	66.7	0.387 (1.897)	10	47.6	**0.049 (6.016)**
≤5 days/week	164	124	75.6		91	55.5	
>5 days/week	109	75	68.8		44	40.4^2^	
Total	294	213	72.4		145	49.3	

^1^As previously reported by Yordanov et al. [29]; ^2^*p* and *χ*^2^ values were according to the univariate analysis; the statistically significant values are shown in bold; ^3^the number was lower than the total number of subjects due to some missing information.

**Table 2:** Risk factors for positive CagA IgG among 294 Bulgarian healthy donors confirmed by logistic regression analysis.

Outcome variable	Exposure (independent) variable	B^1^	SEM^2^	*p*	OR	95% CI^3^
Positive for CagA IgG	Age	0.035	0.011	0.001	1036	1.014–1.057
Positive for CagA IgG	Yoghurt (frequent consumers (>5 days(week) versus the rest of the subjects)	−0.580	0.254	0.022	0.560	0.341–0.921
Positive for CagA IgG	Honey (frequent consumers (>5 days(week) versus the rest of the subjects)	−0.437	0.146	0.003	0.646	0.486–0.859

^1^B: regression coefficient of the logistic regression; ^2^SEM: standard error of the mean of the regression coefficients; ^3^95% CI for the OR (Exp(B)).

**Table 3:** Risk factors for positive H pylori IgG among 294 Bulgarian healthy donors confirmed by logistic regression analysis.

Outcome variable	Exposure (independent) variable	B^1^	SEM^2^	*p*	OR	95% CI^3^
Positive for *H. pylori* IgG	Age	0.048	0.012	<0.001	1.049	1.024–1.075
Positive for *H. pylori* IgG	Honey (frequent consumers (>5 days(week) versus the rest of the subjects)	−0.508	0.182	0.032	0.676	0.473–0.967

^1^B: regression coefficient of the logistic regression; ^2^SEM: standard error of the mean of the regression coefficients; ^3^95% CI for the OR (Exp(B)).
